# Identification of differentially expressed ferroptosis-related genes in abdominal aortic aneurysm: Bioinformatics analysis

**DOI:** 10.3389/fcvm.2022.991613

**Published:** 2022-09-29

**Authors:** Kun Wang, Yancheng Song, Hong Li, Jianshu Song, Shizhong Wang

**Affiliations:** ^1^Department of Cardiovascular Surgery, The Affiliated Hospital of Qingdao University, Qingdao, China; ^2^Department of Gastrointestinal Surgery, The Affiliated Hospital of Qingdao University, Qingdao, China; ^3^Clinical Laboratory, The Affiliated Qingdao Hiser Hospital of Qingdao University, Qingdao, China

**Keywords:** AAA, ferroptosis, bioinformatics analysis, IL-6, gene expression

## Abstract

**Purpose:**

Ferroptosis plays a crucial role in the development and progression of abdominal aortic aneurysm (AAA). The aim of this study was to identify differentially expressed genes associated with ferroptosis in AAA through bioinformatics analysis combined with experimental validation.

**Materials and methods:**

Firstly, the mRNA expression profile datasets GSE57691 and GSE47472 from Gene Expression Omnibus database were screened, and principal component analysis was carried out. Next, the R software (version 4.0.0) was used to analyze potentially differentially expressed genes associated with AAA and ferroptosis. Subsequently, protein–protein interaction analysis, gene ontology enrichment analysis, and Kyoto Encyclopedia of Genes and Genomes pathway enrichment analysis were performed on the selected candidate genes. Finally, quantitative real-time polymerase chain reaction (qRT-PCR) was used to detect the expression levels of the first five selected abnormal ferroptosis-related genes in clinical samples obtained from patients with AAA and healthy controls.

**Results:**

Based on the information contained in the two datasets, a total of 20 differentially expressed ferroptosis-related genes (three upregulated genes and 17 downregulated genes) were selected. Protein–protein interaction analysis demonstrated interaction between these genes, while gene ontology enrichment analysis of ferroptosis genes with differential expression indicated that some enrichment items were associated with oxidative stress. The qRT-PCR results showed that the expression levels of interleukin-6 (IL-6), peroxiredoxin 1 (PRDX1), and stearoyl-CoA desaturase (SCD) were consistent with the bioinformatics prediction results obtained from the mRNA chip.

**Conclusion:**

Bioinformatics analysis identified 20 potential ferroptosis-related differentially expressed genes in AAA. Further verification by qRT-PCR showed that IL-6, PRXD1, and SCD might affect the process of AAA by regulating ferroptosis. Our results might assist in further understanding the pathogenesis of AAA and guiding treatment.

## Introduction

Abdominal aortic aneurysm (AAA) is a cardiovascular disease characterized by segmental progressive dilation of the abdominal aorta (1). The rupture of an aneurysm leads to very serious consequences (2). At present, the main treatment options for AAA are open surgery or endovascular aneurysm repair (3). Epidemiological studies have shown that the incidence of AAA is 1.9–18.5% in males and 0–4.2% in females (4, 5). Risk factors associated with AAA (e.g., sex, aging, smoking, hypertension, and history of coronary heart disease) have been extensively explored (4). An increasing number of studies have also reported that various biological functions, including ferroptosis, autophagy, and inflammation, are involved in the occurrence and development of AAA (1, 6–8). Ferroptosis plays a key role in the pathogenesis of AAA.

The term ferroptosis refers to cell death caused by uncontrolled lipid peroxidation (9). Ferroptosis has been strongly associated with a variety of cardiovascular diseases (6). For example, activating transcription factor 3 (ATF3) might be involved in atherosclerotic plaque formation through ferroptosis (10). In addition, BRD4770 prevents aortic dissection by inhibiting ferroptosis (11). The role of ferroptosis in AAA has been reported; for example, cigarette smoke was shown to induce ferroptosis in vascular smooth muscle cells in AAA (6). However, research on ferroptosis-related genes in AAA is currently limited. The identification of ferroptosis-related genes involved in AAA might provide useful biomarkers and targets for further research on this disease.

Biros et al. published a dataset (GSE57691) that included differentially expressed genes between patients with AAA and healthy individuals (12). They identified 840 and 1,014 differentially expressed genes in small AAA (diameter: ≤55 mm) and large AAA (diameter: >55 mm), respectively. Moreover, some differentially expressed genes were selected for verification. In their previous study, 1,047 differentially expressed genes were identified in carotid artery samples obtained from patients with AAA and aortic samples extracted from organ donors by constructing the dataset GSE47472 (13). Additionally, quantitative polymerase chain reaction (qPCR) was used to demonstrate the differential expression of genes filtered by bioinformatics analysis. In this study, other directions were explored according to the results reported by Biros et al. By combining the GSE57691 and GSE47472 datasets from the Gene Expression Omnibus (GEO) database, we analyzed the differentially expressed genes associated with ferroptosis in AAA. Initially, we identified 20 candidate genes. Subsequently, protein–protein interaction (PPI) and gene ontology (GO) enrichment analyses were performed on the candidate genes. Finally, we identified key genes among the candidate genes and examined their expression levels in clinical samples of AAA.

## Materials and methods

### Ferroptosis-related gene datasets and microarray data

**FIGURE 1:**
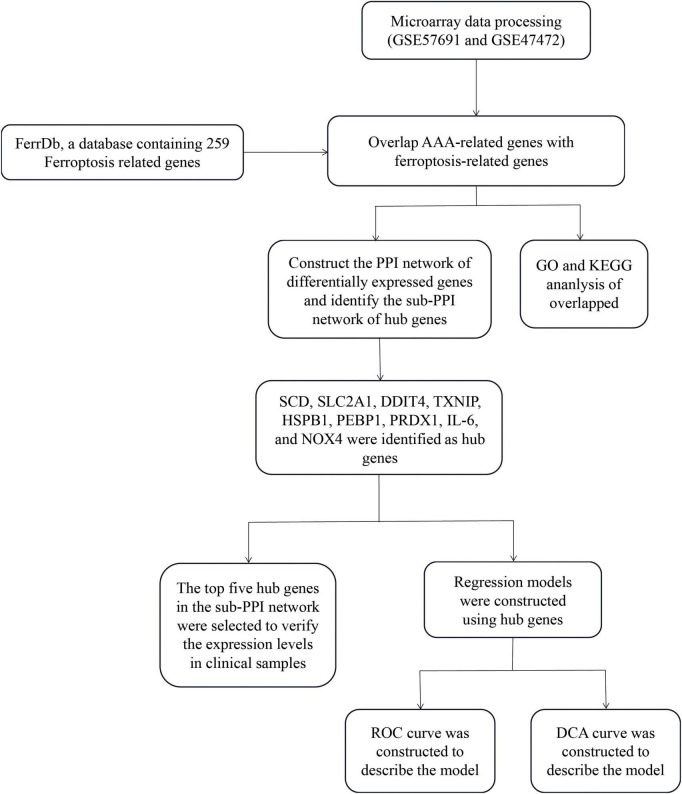
Workflow of data preparation, processing, analysis, and validation.

The GSE57691 dataset was present on the GPL10558 platform (Illumina HumanHT-12 V4.0 expression beadchip) and included 20 patients with small AAA (mean maximum aortic diameter: 54.3 ± 2.3 mm) and 29 patients with large AAA (mean maximum aortic diameter: 68.4 ± 14.3 mm). The GSE47472 dataset was also derived from the GPL10558 platform (Illumina HumanHT-12 V4.0 expression beadchip) and contained carotid artery specimens obtained from 14 patients with AAA (mean maximum aortic diameter: 62.6 ± 18.0 mm). The mRNA expression profiles of these two datasets were obtained from the GEO.^1^ There are 259 genes in the human ferroptosis database.^2^ The overall research process of the present study is shown in Figure 1.

### Analysis of differentially expressed ferroptosis-related genes

To obtain a standardized expression matrix for microarray data, we downloaded information from the dataset and annotated probes according to the annotation files. Principal component analysis was used to test the repeatability of the data in the GSE and SVA R package was used to remove batch effects. Data was standardized using the “Limma” toolkit in the R software (version 4.0.0). A | log_2_ (fold change)| > 0.5 and a *P*-value < 0.05 were used as the criteria for differential gene expression. The heatmap and volcano plot were drawn using the “Heatmap” and “ggplot2” packages in the R software (version 4.0.0).

### Protein–protein interaction and correlation analyses of differentially expressed ferroptosis-related genes

The Cytoscape software (version 3.8.1) and Search Tool for the Retrieval of Interacting Genes (STRING) database^3^ were used to conduct the PPI analysis of differentially expressed ferroptosis-related genes. Firstly, the STRING database was used to construct a PPI network containing the differentially expressed ferroptosis-related genes. The PPI file is then imported into Cytoscape (version 3.8.1) and mapped onto the PPI. Finally, the node degree of each node and the average node degree of each protein in the network were defined to generate the threshold of PPI network nodes, which was used as the screening standard to screen out the proteins with node degree greater than the threshold. Spearman correlation from the “Corrplot” package of the R software was used for correlation analysis of these genes.

### Gene ontology and kyoto encyclopedia of genes and genomes pathway enrichment analyses of ferroptosis-related genes

Gene ontology and KEGG pathway enrichment analyses were performed using the clusterProfiler toolkit in the R software. The main domains of GO analysis are biological process (BP) and molecular function.

### Patients with AAA and healthy individuals

From July 2021 to May 2022, we collected clinical samples from five patients with AAA and five healthy individuals at The Affiliated Hospital of Qingdao University (Qingdao, China); these patients formed the case and control groups, respectively (Table 1). The diagnostic criteria for patients with AAA were an artery diameter >3 cm or artery dilation (1.5-fold change) versus the normal artery size, detected through imaging examination. All participants provided written informed consent for their participation in the study. The study was approved by the medical ethics committee of the hospital.

**TABLE 1:** Demographic and clinical characteristics of the included patients with abdominal aortic aneurysm and controls.

Characteristic	Patients (*n* = 5)	Healthy controls (*n* = 5)
Age, years	50.4 ± 9.07	44.8 ± 1.33
Sex, male:female	4:1	2:3
Hypertension, n (%)	5 (100%)	2 (40%)
Diabetes mellitus, n (%)	2 (40%)	0 (0%)
Hyperlipidemia, n (%)	1 (20%)	0 (0%)
Smoking history, n (%)	3 (60%)	1 (20%)

### RNA extraction and quantitative real-time PCR (qRT-PCR)

The Kz-111-fp high-speed low-temperature grinding instrument (Servicebio, Qingdao, China) and TRIZOL reagent (Vazyme, Qingdao, China) were used to grind the tissues and extract total RNA. Reverse transcription to cDNA was performed according to instructions provided by the manufacturer (SparkJade, Qingdao, China). The 2 × SYBR Green qPCR Mix (SparkJade) was used, and the reaction was run at 94°C for 3 min, 94°C for 10 s, and 60°C for 30 s and ≥40 cycles. All experimental data are presented as the mean values obtained from three independent experiments and were analyzed statistically using the 2^–ΔΔ^ cycle threshold method. Glyceraldehyde-3-phosphate dehydrogenase (GAPDH) was used as internal reference. The primers used for qRT-PCR are listed in Table 2.

**TABLE 2:** Primer sequences used for qRT-PCR.

Primer	Sequences (5′→3′)
GAPDH-F	AAGAAGGTGGTGAAGCAGGC
GAPDH-R	TCCACCACCCAGTTGCTGTA
PRDX1-F	GACTGGGACCCATGAACATTCC
PRDX1-R	TGAACGAGATGCCTTCATCAGC
TXNIP-F	GGGTGTCTGTCTCTGCTCGAA
TXNIP-R	TGGCCATTGGCAAGGTAAGTG
NOX4-F	GTTTCAAAGCTGGTCTGCCATTCTA
NOX4-R	GATGAAGCCCTGCAGAAGCAA
SCD-F	TACCGCTGGCACATCAACTTC
SCD-R	CGGCCTTGGAGACTTTCTTCC

qRT-PCR, quantitative real-time polymerase chain reaction.

### Statistical analysis

The R software (version 4.0.0) was used for the statistical analysis of the bioinformatics data. The Student’s *t*-test was used to evaluate gene expression levels in clinical samples. *P*-values < 0.05 denoted statistically significant differences.

## Results

### Retrospective analysis of differential expression of ferroptosis-related genes in abdominal aortic aneurysm

**FIGURE 2:**
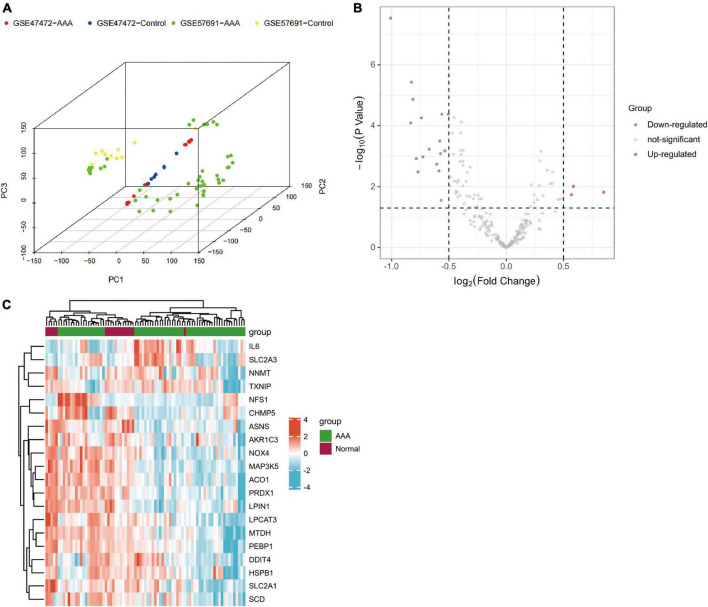
Differentially expressed ferroptosis-related genes in AAA and healthy samples. **(A)** Results of the PCA. In the PCA picture, AAA in GSE47472 is marked in red, control in GSE47472 is marked in blue, AAA in GSE57691 is marked in green and control in 8 GSE57691 is marked in yellow. **(B)** Volcano plot of differentially expressed ferroptosis-related genes. Significantly upregulated and downregulated genes are represented by red and blue dots, respectively. Criteria used for the identification of differences: P<0.05 and | log FC| > 0.5. **(C)** Heatmap of 20 differentially expressed ferroptosis-related genes in AAA and healthy samples. AAA, abdominal aortic aneurysm; FC, fold change; PCA, principal component analysis.

**FIGURE 3:**
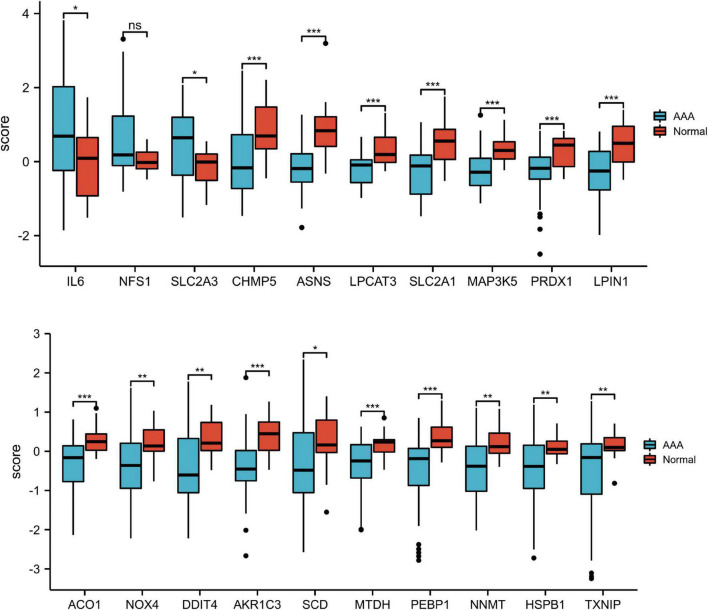
Boxplot of 20 differentially expressed ferroptosis-related genes in AAA and healthy samples. The “score” on the *Y*-axis represents relative gene expression. The blue and red boxes represent AAA and healthy samples, respectively. *P < 0.05; **P < 0.01; ***P < 0.001. AAA, abdominal aortic aneurysm; FC, fold change; ns, non-significant; PCA, principal component analysis.

Firstly, principal component analysis (PCA) was performed on the two groups of data (Supplementary Figure 1). Then we eliminated the batch effect in PCA (Figure 2A). Subsequently, utilizing the adjusted *P*-value < 0.05 and | log_2_ (fold change)| > 0.5 as the standards, we selected 20 genes from the 259 ferroptosis-related genes (three upregulated genes and 17 downregulated genes) (Table 3). The 20 differentially expressed ferroptosis-related genes between the AAA and normal groups identified in the GSE57691 and GSE47472 databases are shown in volcano (Figure 2B) and heatmap (Figure 2C) plots. The expression patterns of 20 candidate genes in AAA and normal samples are shown in a box plot (Figure 3). Of the three upregulated genes, interleukin-6 (IL-6) and solute carrier family 2 member 3 (SLC2A3) had statistically significant changes in expression. Among the downregulated genes, charged multivesicular body protein 5 (CHMP5), asparagine synthetase (ASNS), aconitase 1 (ACO1), metadherin (MTDH), etc. exhibited significant changes in expression.

**TABLE 3:** The 20 differentially expressed ferroptosis-related genes identified in AAA samples compared with healthy samples.

Gene symbol	LogFC	Changes	*P*-value	Adjusted *P*-value
IL-6	0.850885624	Up	0.15267891	0.119686661
NFS1	0.569253374	Up	0.018340251	0.134909235
SLC2A3	0.586111099	Up	0.00975004	0.08830804
CHMP5	−0.785718442	Down	0.001204301	0.022131658
ASNS	−1.009571559	Down	2.96E-08	1.48E-05
LPCAT3	−0.503984405	Down	4.23E-05	0.002450013
SLC2A1	−0.827933287	Down	3.70E-06	0.00046298
MAP3K5	−0.562187827	Down	4.27E-05	0.002450013
PRDX1	−0.534885914	Down	0.000668838	0.015070277
LPIN1	−0.741872954	Down	5.58E-05	0.002924138
ACO1	−0.582423279	Down	0.000318159	0.009160088
NOX4	−0.607734917	Down	0.001827326	0.029096612
DDIT4	−0.72853054	Down	0.001056212	0.020396489
AKR1C3	−0.814884493	Down	1.35E-05	0.001190744
SCD	−0.568083484	Down	0.027865333	0.17916661
MTDH	−0.574434868	Down	0.000824557	0.017314976
PEBP1	−0.834702749	Down	8.20E-05	0.00375861
NNMT	−0.673897508	Down	0.000592542	0.01392957
HSPB1	−0.589272465	Down	0.003014269	0.040507169
TXNIP	−0.770900911	Down	0.003280469	0.042649467

AAA, abdominal aortic aneurysm.

### Protein–protein interaction network and identification of hub genes among the candidate ferroptosis-related genes

**FIGURE 4:**
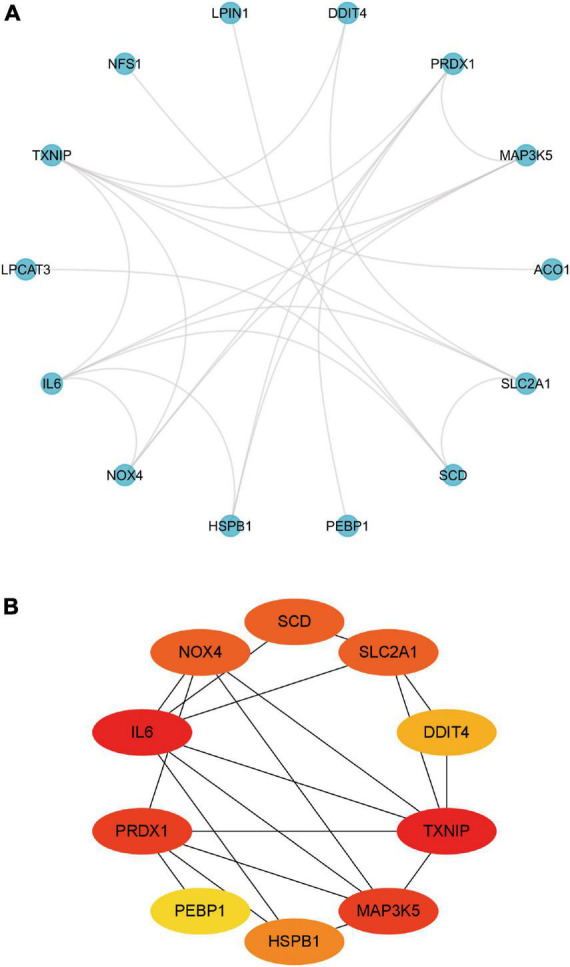
PPI analysis of candidate differentially expressed ferroptosis-related genes. **(A)** PPI networks of candidate genes. **(B)** PPI subnetwork of the top 10 hub candidate genes. PPI, protein–protein interaction.

The PPI analysis demonstrated that candidate genes interacted with each other (Supplementary Figure 2). Candidate genes that did not interact according to the predicted results were ignored (Figure 4A). In the PPI network, the top 10 scoring genes constitute the hub framework (Figure 4B). The hub ferroptosis-related genes were stearoyl-CoA desaturase (SCD), SLC2A1, DNA damage inducible transcript 4 (DDIT4), thioredoxin interacting protein (TXNIP), heat shock protein family B (small) member 1 (HSPB1), phosphatidylethanolamine binding protein 1 (PEBP1), peroxiredoxin 1 (PRDX1), IL-6, and NADPH oxidase 4 (NOX4).

### Gene ontology enrichment analysis of candidate ferroptosis-related genes

**FIGURE 5:**
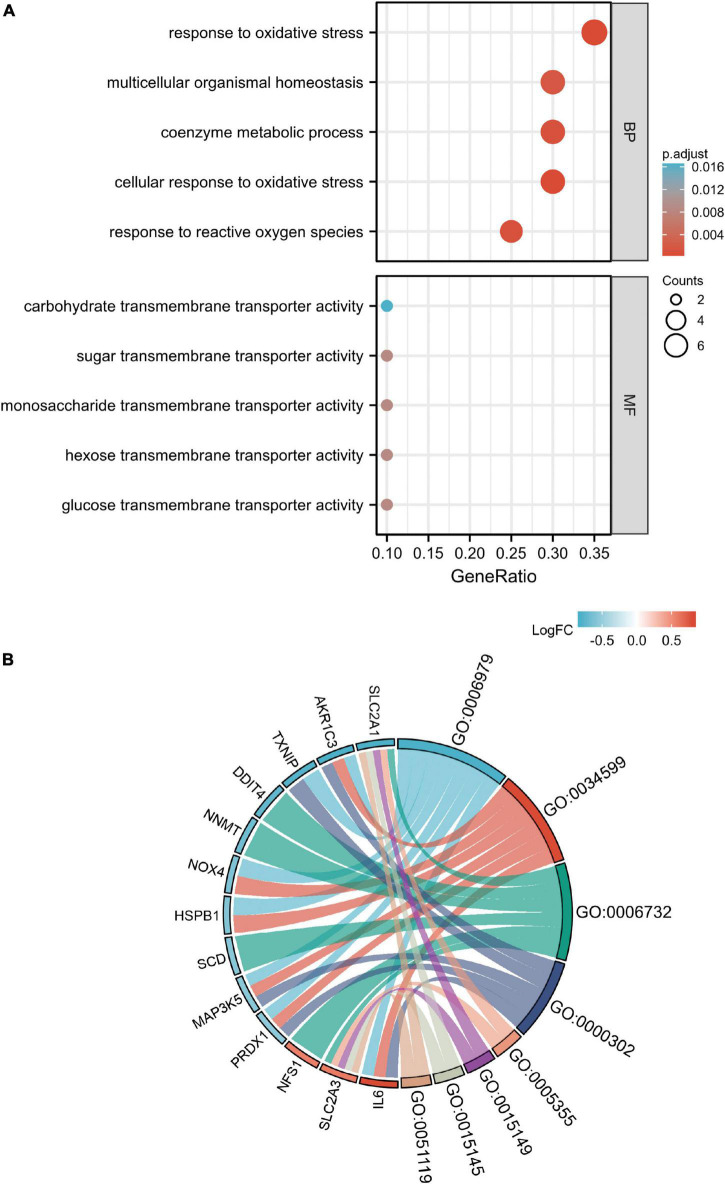
Gene ontology (GO) enrichment analysis of 20 differentially expressed ferroptosis-related genes. Bubble diagram of GO enrichment term **(A)** and chord diagram of GO enrichment term **(B)**. Some candidate genes are not shown in chord diagram because the enrichment is too scattered.

The results of the GO enrichment analysis (Figures 5A,B) suggested that the main enriched terms were associated with response to oxidative stress, multicellular organismal homeostasis, coenzyme metabolic process, cellular response to oxidative stress, and response to reactive oxygen species (BP). However, the enrichment of carbohydrate transmembrane transporter activity, sugar transmembrane transporter activity, and other molecular functions was limited. The contents related to cellular components were not enriched. We also conducted a KEGG pathway enrichment analysis that did not yield significant results.

### Performance of candidate ferroptosis-related genes

**FIGURE 6:**
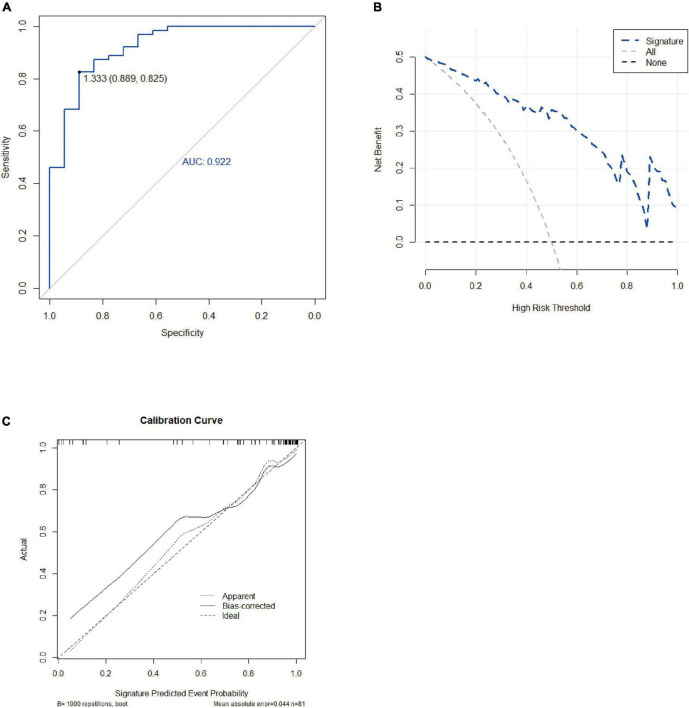
Testing of the model. ROC, DCA, and calibration curve were used to detect 10 hub genes. **(A)** ROC curves of the ferroptosis-related gene signature that was a candidate for differential expression. **(B)** DCA of the candidate ferroptosis-related gene signature. **(C)** Calibration curve of the model. DCA, decision curve analysis; ROC, receiver operating characteristic.

The receiver operating characteristic (ROC) curve was used to describe that the logistic regression model constructed with 10 hub genes has excellent sensitivity for the diagnosis of AAA. The area under the curve value of the candidate ferroptosis-related genes was 0.922 (Figure 6A); higher values indicate higher accuracy of the logistic regression model constructed by the 10 hub genes for predicting the development of AAA.

In the logistic regression model constructed using 10 hub genes, the decision curve analysis described that the net benefit could be higher than the marked line in all threshold probability intervals (0 to 1) by intervening hub genes (Figure 6B). This suggests that the logistic regression model constructed using these 10 hub genes has clinical significance. Therefore, intervention using this logistic regression model may improve the prognosis of patients with AAA. However, due to the lack of existing clinical interventions for comparison, it is difficult to examine its potential superiority. The calibration curve showed a good fit and supported the establishment of the model (Figure 6C).

### Validation of differentially expressed ferroptosis-related candidate genes in abdominal aortic aneurysm clinical samples

**FIGURE 7:**
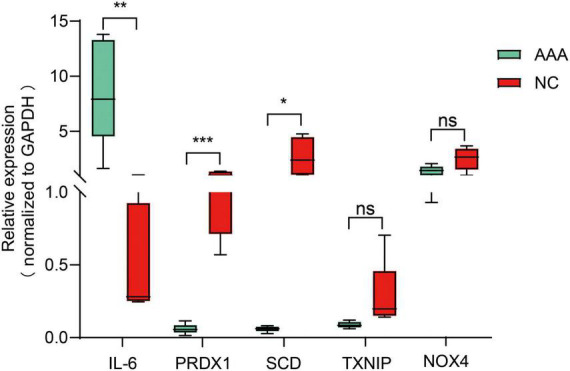
Verification of the expression of the first five hub candidate genes in clinical samples: IL-6, PRDX1, SCD, TXNIP, and NOX4. P-values were calculated using the Student’s *t*-test. *P < 0.05; **P < 0.01; ***P < 0.001. IL-6, interleukin-6; PRDX1, peroxiredoxin 1; SCD, stearoyl-CoA desaturase; TXNIP, thioredoxin interacting protein; NOX4, NADPH oxidase 4; ns, non-significant.

To further examine the expression of differentially expressed ferroptosis-related genes in AAA, we examined the top five differentially expressed candidate genes and detected their expression in clinical samples by qRT-PCR (Figure 7). Our results showed that IL-6, SCD, and PRDX1 in the clinical samples of AAA demonstrated statistically significant changes in expression. Although TXNIP and NOX4 showed a trend consistent with our expectations, the observed change was not statistically significant.

## Discussion

Abdominal aortic aneurysm is a cardiovascular disease characterized by aortic dilation. This leads to weakening of the wall of the aorta and an eventual rupture. In studies, the incidence of AAA rupture was positively correlated with the diameter of aortic wall enlargement, and AAA rupture was an important cause of death (14). Currently, inflammation, oxidative stress, smooth muscle cell apoptosis, and extracellular matrix degradation have been associated with the development of AAA (15). Inflammation is a characteristic pathological event related to AAA (16). In a recent study, Ni et al. found that Notch1 is involved in the development of AAA by regulating NLR family pyrin domain containing 3 (NLRP3) inflammasome and macrophage activation (16). Nuclear factor-κB (NF-κB) signaling, a classic pathway that regulates inflammation, also promotes inflammation in AAA (17). Lin et al. reported that long non-coding RNA SRY-box transcription factor 2 (Sox2) overlapping transcript plays an important role in the oxidative stress response of vascular smooth muscle cells in AAA (18). This also provides evidence that oxidative stress is involved in the occurrence and development of AAA. Studies discovered traces of ferroptosis in the complex pathological process of AAA; iron overload promotes the progression of AAA (19). Moreover, the effect of cigarette extract on promoting smooth muscle cell death in AAA was mitigated by ferroptosis-specific inhibitors (20). This suggests that ferroptosis is involved in AAA; nevertheless, its specific role remains obscure.

Biological process analysis showed that the candidate ferroptosis-related genes were mainly enriched in oxidative stress. This finding appears reasonable because oxidative stress has been suggested as a possible key factor in the development of ferroptosis (21–23). Ferroptosis involves abnormal iron homeostasis and lipid peroxidation metabolism. The metabolic disorder of cells catalyzed by iron destroys the redox balance and eventually leads to cell death. Ferroptosis has been investigated as a key factor in numerous cardiovascular diseases (24). In atherosclerosis, it has been linked to the regulation of nuclear factor erythroid 2-related factor 2-kelch like ECH associated protein 1 (NRF2-KEAP1) and p53 (25–30). Recently, it has been reported that BRD4770 delays the progression of aortic dissection by inhibiting ferroptosis (11). However, the specific role of ferroptosis in the occurrence and development of AAA remains to be further investigated. Our research provides possible directions for the exploration of ferroptosis in AAA. Through bioinformatics analysis, we identified 20 genes that might play a role in ferroptosis in AAA. GO analysis showed that these genes were mainly involved in oxidative stress response, and iron homeostasis has been associated with oxidative stress (21). An abnormal aerobic environment controls the toxicity of iron and leads to ferroptosis (21). Nonetheless, it has also been reported that oxidative stress is involved in the occurrence and development of AAA (31–34). Based on this evidence, the present research might provide a reference for ferroptosis induced by peroxide stress response in AAA. To the best of our knowledge, this direction is rarely explored.

In clinical samples, we found that the expression levels of IL-6, PRDX1, and SCD were consistent with the biological information of the mRNA chip. IL-6 is involved in inflammatory processes in numerous diseases (35). It is highly expressed in AAA tissues, induces leukocyte aggregation, and promotes AAA inflammation by regulating the expression of chemokines (36–38). Wang et al. recently reported that IL-12p35 regulates IL-6 through the signal transducer and activator of transcription 4 (STAT4) pathway in the inflammatory process of AAA. They also confirmed that IL-6 plays a role in inflammation in AAA (39). However, another study showed that IL-6 has a limited contribution to inflammation in AAA (37). Hence, the function of IL-6 in AAA might not be limited to its role in inflammation. Sheng et al. found that IL-6 regulates ferroptosis through the Mir-10a-5p/IL-6R axis (40). Moreover, Zhang et al. suggested that the effect of elabela on ferroptosis was associated with IL-6 (41). This evidence implies that IL-6 regulates ferroptosis in cells. However, there are no distinct reports concerning the involvement of IL-6 in ferroptosis in AAA. Our study might serve as a basis for further research on the regulation of ferroptosis in AAA by IL-6; nevertheless, further follow-up experiments are warranted to confirm our hypothesis. PRDX1 was originally identified as a peroxide-scavenging enzyme; however, its functions are not limited to antioxidation, molecular chaperones, and signal transduction (42–44). Recent studies suggested that PRDX1 is involved in oxidative stress-induced ferroptosis in cells. Lovatt et al. demonstrated that loss of PRDX1 induced ferroptosis in corneal endothelial cells, and this process was associated with PRDX1-mediated lipid peroxidation (45). This evidence supports our current conclusion that PRDX1 is resistant to ferroptosis; nevertheless, the specific mechanism involved in this process is unclear. Moreover, PRDX1 is a biomarker for AAA (46). Therefore, our conclusion might provide a basis for the further exploration of the relationship between ferroptosis and PRDX1 in AAA. It has been shown that SCD controls the quantity of monounsaturated fatty acids, which in turn are involved in cell growth, metabolism, and signal transduction (47). Research has revealed that SCD is highly expressed in pancreatic and bladder cancers, and protects cancer cells from ferroptosis (48, 49). Thus far, the roles of SCD have not been studied in AAA. Our results might provide directions for additional research on these topics.

The present study has some limitations. Firstly, the clinical sample size included in this investigation was small. Secondly, although we determined the expression levels of differentially expressed ferroptosis-related genes in AAA, we did not discuss the specific mechanisms of these candidate genes in animal models or AAA cells. These mechanisms need to be examined by further experiments in the future.

## Conclusion

In summary, we identified 20 potential genes associated with ferroptosis in AAA through bioinformatics analysis. Among them, IL-6, PRDX1, and SCD might participate in the occurrence and development of AAA by regulating ferroptosis. The present findings might enhance our understanding of AAA and, to some extent, guide treatment.

## Data availability statement

Publicly available datasets were analyzed in this study. This data can be found here: Gene Expression Omnibus database, GSE57691 and GSE47472.

## Ethics statement

The studies involving human participants were reviewed and approved by Medical Ethics Committee of Affiliated Hospital of Qingdao University. The patients/participants provided their written informed consent to participate in this study.

## Author contributions

KW: investigation, experimental verification, writing—original draft, and visualization. YS: investigation and visualization. HL and JS: investigation. SW: conceptualization, resources, writing—review and editing, supervision, and project administration. All authors contributed to the article and approved the submitted version.

## Footnotes

1
http://www.ncbi.nlm.nih.gov/geo/

2
http://www.zhounan.org/ferrdb/legacy/operations/download.html

3
https://string-db.org/

## Supplementary material

Supplementary Figure 1 PCA of GSE57691 and GSE47472 before removing the batch effecte. PCA, principal component analysis.

Supplementary Figure 2 Complete PPI network of 20 differentially expressed ferroptosis-related genes. PPI, protein–protein interaction.

## References

[1] Wang YD Liu ZJ Ren J Xiang MX. Pharmacological therapy of abdominal aortic aneurysm: an update. *Curr Vasc Pharmacol.* (2018) 16:114–24. 10.2174/1570161115666170413145705 28412911

[2] Tchana-Sato V Sakalihasan N Defraigne JO. Ruptured abdominal aortic aneurysm. *Rev Med Liege.* (2018) 73:296–9. 29926569

[3] Abraham G Vijayan M Gopalakrishnan N Shroff S Amalorpavanathan J Yuvaraj A State of deceased donor transplantation in India: a model for developing countries around the world. *World J Transplant.* (2016) 6:331–5. 10.5500/wjt.v6.i2.331 27358778 PMC4919737

[4] Altobelli E Rapacchietta L Profeta VF Fagnano R. Risk factors for abdominal aortic aneurysm in population-based studies: a systematic review and meta-analysis. *Int J Environ Res Public Health.* (2018) 15:2805. 10.3390/ijerph15122805 30544688 PMC6313801

[5] Ullery BW Hallett RL Fleischmann D. Epidemiology and contemporary management of abdominal aortic aneurysms. *Abdom Radiol.* (2018) 43:1032–43. 10.1007/s00261-017-1450-7 29313113

[6] Zhang Y Xin L Xiang M Shang C Wang Y Wang Y The molecular mechanisms of ferroptosis and its role in cardiovascular disease. *Biomed Pharmacother.* (2022) 145:112423. 10.1016/j.biopha.2021.112423 34800783

[7] Wang L Liu S Pan B Cai H Zhou H Yang P The role of autophagy in abdominal aortic aneurysm: protective but dysfunctional. *Cell Cycle.* (2020) 19:2749–59. 10.1080/15384101.2020.1823731 32960711 PMC7714418

[8] Yuan Z Lu Y Wei J Wu J Yang J Cai Z. Abdominal aortic aneurysm: roles of inflammatory cells. *Front Immunol.* (2020) 11:609161. 10.3389/fimmu.2020.609161 33613530 PMC7886696

[9] Jiang X Stockwell BR Conrad M. Ferroptosis: mechanisms, biology and role in disease. *Nat Rev Mol Cell Biol.* (2021) 22:266–82. 10.1038/s41580-020-00324-8 33495651 PMC8142022

[10] Ouyang S You J Zhi C Li P Lin X Tan X Ferroptosis: the potential value target in atherosclerosis. *Cell Death Dis.* (2021) 12:782. 10.1038/s41419-021-04054-3 34376636 PMC8355346

[11] Chen Y Yi X Huo B He Y Guo X Zhang Z BRD4770 functions as a novel ferroptosis inhibitor to protect against aortic dissection. *Pharmacol Res.* (2022) 177:106122. 10.1016/j.phrs.2022.106122 35149187

[12] Biros E Gäbel G Moran CS Schreurs C Lindeman JH Walker PJ Differential gene expression in human abdominal aortic aneurysm and aortic occlusive disease. *Oncotarget.* (2015) 6:12984–96. 10.18632/oncotarget.3848 25944698 PMC4536993

[13] Biros E Moran CS Rush CM Gäbel G Schreurs C Lindeman JH Differential gene expression in the proximal neck of human abdominal aortic aneurysm. *Atherosclerosis.* (2014) 233:211–8. 10.1016/j.atherosclerosis.2013.12.017 24529146

[14] Filardo G Powell JT Martinez MA Ballard DJ. Surgery for small asymptomatic abdominal aortic aneurysms. *Cochrane Database Syst Rev.* (2015) 2015:Cd001835. 10.1002/14651858.CD001835.pub4 25927098 PMC6464801

[15] Kuivaniemi H Ryer EJ Elmore JR Tromp G. Understanding the pathogenesis of abdominal aortic aneurysms. *Expert Rev Cardiovasc Ther.* (2015) 13:975–87. 10.1586/14779072.2015.1074861 26308600 PMC4829576

[16] Ni XQ Zhang YR Jia LX Lu WW Zhu Q Ren JL Inhibition of Notch1-mediated inflammation by intermedin protects against abdominal aortic aneurysm via PI3K/Akt signaling pathway. *Aging.* (2021) 13:5164–84. 10.18632/aging.202436 33535178 PMC7950288

[17] Ren J Han Y Ren T Fang H Xu X Lun Y AEBP1 promotes the occurrence and development of abdominal aortic aneurysm by modulating inflammation via the NF-κB pathway. *J Atheroscler Thromb.* (2020) 27:255–70. 10.5551/jat.49106 31462616 PMC7113137

[18] Lin H You B Lin X Wang X Zhou D Chen Z Silencing of long non-coding RNA Sox2ot inhibits oxidative stress and inflammation of vascular smooth muscle cells in abdominal aortic aneurysm via microRNA-145-mediated Egr1 inhibition. *Aging.* (2020) 12:12684–702. 10.18632/aging.103077 32629426 PMC7377859

[19] Sawada H Hao H Naito Y Oboshi M Hirotani S Mitsuno M Aortic iron overload with oxidative stress and inflammation in human and murine abdominal aortic aneurysm. *Arterioscler Thromb Vasc Biol.* (2015) 35:1507–14. 10.1161/ATVBAHA.115.305586 25882069

[20] Sampilvanjil A Karasawa T Yamada N Komada T Higashi T Baatarjav C Cigarette smoke extract induces ferroptosis in vascular smooth muscle cells. *Am J Physiol Heart Circ Physiol.* (2020) 318:H508–18. 10.1152/ajpheart.00559.2019 31975626

[21] Galaris D Barbouti A Pantopoulos K. Iron homeostasis and oxidative stress: an intimate relationship. *Biochim Biophys Acta Mol Cell Res.* (2019) 1866:118535. 10.1016/j.bbamcr.2019.118535 31446062

[22] Weiland A Wang Y Wu W Lan X Han X Li Q Ferroptosis and its role in diverse brain diseases. *Mol Neurobiol.* (2019) 56:4880–93. 10.1007/s12035-018-1403-3 30406908 PMC6506411

[23] Liu J Kuang F Kroemer G Klionsky DJ Kang R Tang D. Autophagy-dependent ferroptosis: machinery and regulation. *Cell Chem Biol.* (2020) 27:420–35. 10.1016/j.chembiol.2020.02.005 32160513 PMC7166192

[24] Li J Cao F Yin HL Huang ZJ Lin ZT Mao N Ferroptosis: past, present and future. *Cell Death Dis.* (2020) 11:88. 10.1038/s41419-020-2298-2 32015325 PMC6997353

[25] Klóska D Kopacz A Piechota-Polańczyk A Neumayer C Huk I Dulak J Biliverdin reductase deficiency triggers an endothelial-to-mesenchymal transition in human endothelial cells. *Arch Biochem Biophys.* (2019) 678:108182. 10.1016/j.abb.2019.108182 31704097

[26] Zhao Y Lu J Mao A Zhang R Guan S. Autophagy inhibition plays a protective role in ferroptosis induced by alcohol via the p62-Keap1-Nrf2 pathway. *J Agric Food Chem.* (2021) 69:9671–83. 10.1021/acs.jafc.1c03751 34388345

[27] Chen CJ Huang HS Chang WC. Inhibition of arachidonate metabolism in human epidermoid carcinoma a431 cells overexpressing phospholipid hydroperoxide glutathione peroxidase. *J Biomed Sci.* (2002) 9:453–9. 10.1007/BF02256540 12218361

[28] Jiang L Kon N Li T Wang SJ Su T Hibshoosh H Ferroptosis as a p53-mediated activity during tumour suppression. *Nature.* (2015) 520:57–62. 10.1038/nature14344 25799988 PMC4455927

[29] Gao M Monian P Quadri N Ramasamy R Jiang X. Glutaminolysis and transferrin regulate ferroptosis. *Mol Cell.* (2015) 59:298–308. 10.1016/j.molcel.2015.06.011 26166707 PMC4506736

[30] Ou Y Wang SJ Li D Chu B Gu W. Activation of SAT1 engages polyamine metabolism with p53-mediated ferroptotic responses. *Proc Natl Acad Sci U.S.A.* (2016) 113:E6806–12. 10.1073/pnas.1607152113 27698118 PMC5098629

[31] Sánchez-Infantes D Nus M Navas-Madroñal M Fité J Pérez B Barros-Membrilla AJ Oxidative stress and inflammatory markers in abdominal aortic aneurysm. *Antioxidants.* (2021) 10:602. 10.3390/antiox10040602 33919749 PMC8070751

[32] Gurung R Choong AM Woo CC Foo R Sorokin V. Genetic and epigenetic mechanisms underlying vascular smooth muscle cell phenotypic modulation in abdominal aortic aneurysm. *Int J Mol Sci.* (2020) 21:6334. 10.3390/ijms21176334 32878347 PMC7504666

[33] Emeto TI Moxon JV Au M Golledge J. Oxidative stress and abdominal aortic aneurysm: potential treatment targets. *Clin Sci.* (2016) 130:301–15. 10.1042/CS20150547 26814202

[34] Meital LT Windsor MT Perissiou M Schulze K Magee R Kuballa A Omega-3 fatty acids decrease oxidative stress and inflammation in macrophages from patients with small abdominal aortic aneurysm. *Sci Rep.* (2019) 9:12978. 10.1038/s41598-019-49362-z 31506475 PMC6736886

[35] Tanaka T Narazaki M Kishimoto T. IL-6 in inflammation, immunity, and disease. *Cold Spring Harb Perspect Biol.* (2014) 6:a016295. 10.1101/cshperspect.a016295 25190079 PMC4176007

[36] Akerman AW Stroud RE Barrs RW Grespin RT McDonald LT LaRue RAC Elevated wall tension initiates interleukin-6 expression and abdominal aortic dilation. *Ann Vasc Surg.* (2018) 46:193–204. 10.1016/j.avsg.2017.10.001 29107003 PMC5894101

[37] Nishihara M Aoki H Ohno S Furusho A Hirakata S Nishida N The role of IL-6 in pathogenesis of abdominal aortic aneurysm in mice. *PLoS One.* (2017) 12:e0185923. 10.1371/journal.pone.0185923 28982132 PMC5628902

[38] Shteinberg D Halak M Shapiro S Kinarty A Sobol E Lahat N Abdominal aortic aneurysm and aortic occlusive disease: a comparison of risk factors and inflammatory response. *Eur J Vasc Endovasc Surg.* (2000) 20:462–5. 10.1053/ejvs.2000.1210 11112466

[39] Wang L Hu C Dong Y Dai F Xu Y Dai Y Silencing IL12p35 promotes angiotensin ii-mediated abdominal aortic aneurysm through activating the STAT4 pathway. *Mediators Inflamm.* (2021) 2021:9450843. 10.1155/2021/9450843 34354545 PMC8331298

[40] Bin S Xin L Lin Z Jinhua Z Rui G Xiang Z. Targeting miR-10a-5p/IL-6R axis for reducing IL-6-induced cartilage cell ferroptosis. *Exp Mol Pathol.* (2021) 118:104570. 10.1016/j.yexmp.2020.104570 33166496

[41] Zhang Z Tang J Song J Xie M Liu Y Dong Z Elabela alleviates ferroptosis, myocardial remodeling, fibrosis and heart dysfunction in hypertensive mice by modulating the IL-6/STAT3/GPX4 signaling. *Free Radic Biol Med.* (2022) 181:130–42. 10.1016/j.freeradbiomed.2022.01.020 35122997

[42] Barranco-Medina S Lázaro JJ Dietz KJ. The oligomeric conformation of peroxiredoxins links redox state to function. *FEBS Lett.* (2009) 583:1809–16. 10.1016/j.febslet.2009.05.029 19464293

[43] Neumann CA Cao J Manevich Y. Peroxiredoxin 1 and its role in cell signaling. *Cell Cycle.* (2009) 8:4072–8. 10.4161/cc.8.24.10242 19923889 PMC7161701

[44] Rhee SG Woo HA Kang D. The role of peroxiredoxins in the transduction of H(2)O(2) Signals. *Antioxid Redox Signal.* (2018) 28:537–57. 10.1089/ars.2017.7167 28587524

[45] Lovatt M Adnan K Kocaba V Dirisamer M Peh GSL Mehta JS. Peroxiredoxin-1 regulates lipid peroxidation in corneal endothelial cells. *Redox Biol.* (2020) 30:101417. 10.1016/j.redox.2019.101417 31901729 PMC6948265

[46] Martinez-Pinna R Ramos-Mozo P Madrigal-Matute J Blanco-Colio LM Lopez JA Calvo E Identification of peroxiredoxin-1 as a novel biomarker of abdominal aortic aneurysm. *Arterioscler Thromb Vasc Biol.* (2011) 31:935–43. 10.1161/ATVBAHA.110.214429 21273562

[47] Kikuchi K Tsukamoto H. Stearoyl-CoA desaturase and tumorigenesis. *Chem Biol Interact.* (2020) 316:108917. 10.1016/j.cbi.2019.108917 31838050 PMC6952240

[48] Tesfay L Paul BT Konstorum A Deng Z Cox AO Lee J Stearoyl-CoA desaturase 1 protects ovarian cancer cells from ferroptotic cell death. *Cancer Res.* (2019) 79:5355–66. 10.1158/0008-5472.CAN-19-0369 31270077 PMC6801059

[49] Ye Z Zhuo Q Hu Q Xu X Mengqi L Zhang Z FBW7-NRA41-SCD1 axis synchronously regulates apoptosis and ferroptosis in pancreatic cancer cells. *Redox Biol.* (2021) 38:101807. 10.1016/j.redox.2020.101807 33271455 PMC7710650

