# Supplementary figures and images for: Identification of differentially expressed ferroptosis-related genes in abdominal aortic aneurysm: Bioinformatics analysis
Source: Front Cardiovasc Med. 2022 Sep 29;9:991613. doi: 10.3389/fcvm.2022.991613 (PMC9558826; doi:10.3389/fcvm.2022.991613)

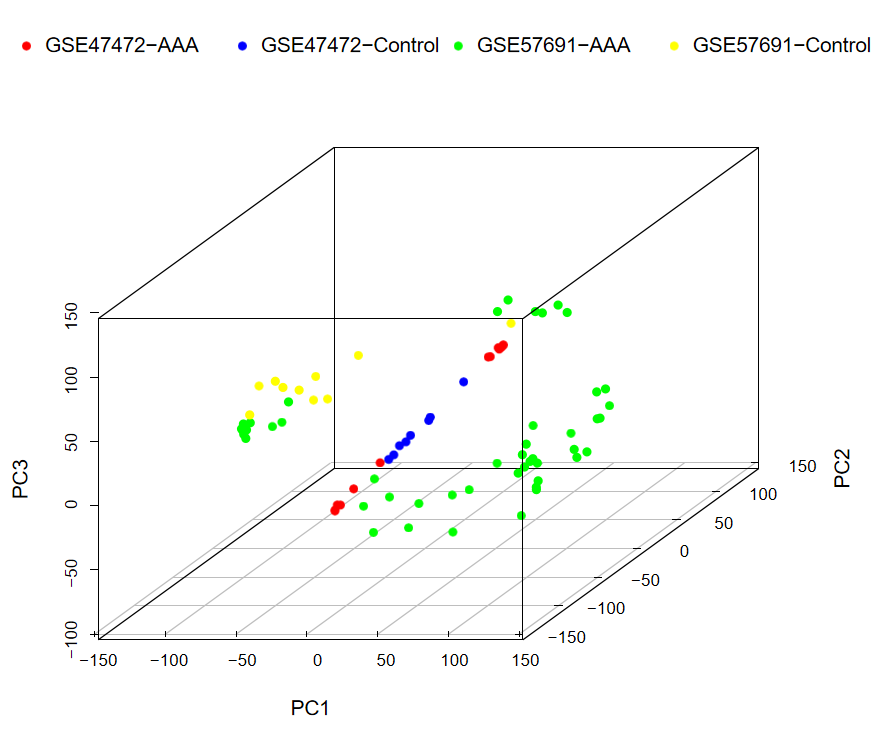

Supplement: Supplementary Figure 1 — PCA of GSE57691 and GSE47472 before removing the batch effecte. PCA, principal component analysis. [file Image_1.PNG]

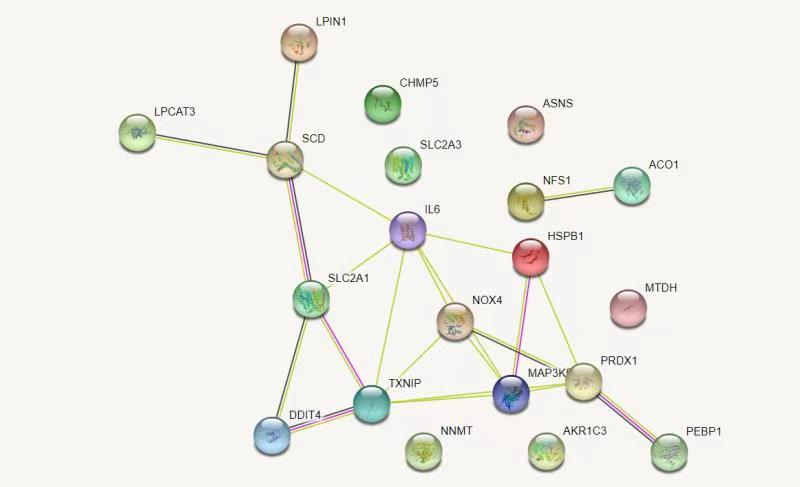

Supplement: Supplementary Figure 2 — Complete PPI network of 20 differentially expressed ferroptosis-related genes. PPI, protein–protein interaction. [file Image_2.JPEG]
